# Hippocampal changes produced by overexpression of the human *CHRNA5/A3/B4* gene cluster may underlie cognitive deficits rescued by nicotine in transgenic mice

**DOI:** 10.1186/s40478-014-0147-1

**Published:** 2014-11-11

**Authors:** Susanna Molas, Thomas Gener, Jofre Güell, Mairena Martín, Inmaculada Ballesteros-Yáñez, Maria V Sanchez-Vives, Mara Dierssen

**Affiliations:** Cellular and Systems Neurobiology, Systems Biology Program, Centre for Genomic Regulation (CRG), Barcelona Biomedical Research Park (PRBB) building, Room 522.04; C/ Dr. Aiguader 88, E-08003 Barcelona, Spain; Universitat Pompeu Fabra (UPF), E-08003 Barcelona, Spain; Centro de Investigación Biomédica en Red de Enfermedades Raras (CIBERER), E-08003 Barcelona, Spain; Department of Inorganic, Organic Chemistry and Biochemistry, Faculty of Medicine, Centro Regional de Investigaciones Biomédicas (CRIB), University of Castilla la Mancha, E-13071 Ciudad Real, Spain; Department of Inorganic, Organic Chemistry and Biochemistry, Faculty of Chemical Sciences and Techonologies, CRIB, University of Castilla la Mancha, E-13071 Ciudad Real, Spain; Institut d’Investigacions Biomèdiques August Pi i Sunyer (IDIBAPS), E-08036 Barcelona, Spain; Institució Catalana de Recerca i Estudis Avançats (ICREA), E-08010 Barcelona, Spain

**Keywords:** *CHRNA5/A3/B4*, Cognition, Hippocampus, Neuroplasticity, Nicotine, Tobacco addiction

## Abstract

Addiction involves long-lasting maladaptive changes including development of disruptive drug-stimuli associations. Nicotine-induced neuroplasticity underlies the development of tobacco addiction but also, in regions such as the hippocampus, the ability of this drug to enhance cognitive capabilities. Here, we propose that the genetic locus of susceptibility to nicotine addiction, the *CHRNA5/A3/B4* gene cluster, encoding the α5, α3 and β4 subunits of the nicotinic acetylcholine receptors (nAChRs), may influence nicotine-induced neuroadaptations. We have used transgenic mice overexpressing the human cluster (Tg*CHRNA5/A3/B4*) to investigate hippocampal structure and function in genetically susceptible individuals. Tg*CHRNA5/A3/B4* mice presented a marked reduction in the dendrite complexity of CA1 hippocampal pyramidal neurons along with an increased dendritic spine density. In addition, Tg*CHRNA5/A3/B4* exhibited increased VGLUT1/VGAT ratio in the CA1 region, suggesting an excitatory/inhibitory imbalance. These hippocampal alterations were accompanied by a significant impairment in short-term novelty recognition memory. Interestingly, chronic infusion of nicotine (3.25 mg/kg/d for 7 d) was able to rescue the reduced dendritic complexity, the excitatory/inhibitory imbalance and the cognitive impairment in Tg*CHRNA5/A3/B4*. Our results suggest that chronic nicotine treatment may represent a compensatory strategy in individuals with altered expression of the *CHRNA5/A3/B4* region.

**Electronic supplementary material:**

The online version of this article (doi:10.1186/s40478-014-0147-1) contains supplementary material, which is available to authorized users.

## Introduction

It is now generally accepted that addiction represents a neuropathology of learning and memory [1]. Drugs of abuse, including nicotine, induce neuroplasticity in areas involved in cognitive function, such as the hippocampus, a neuroadaptation assumed to contribute to the development of addiction by favoring associative memories [2-6]. These neuroadaptations occur at the level of neuronal structure and synaptic strength and also underlie the effects of the drug on cognitive performance [7-11]. In the case of nicotine addiction, two of the most common withdrawal symptoms are both changes in affection and cognition, which, during periods of abstinence from smoking, predict relapse [12]. Nicotinic acetylcholine receptors (nAChRs) are the main targets of nicotine. They also play a significant role in cognition and its disruption has been demonstrated in numerous psychiatric and neurological disorders that present cognitive alterations, including schizophrenia [13], attention deficit hyperactivity disorder [14] or Alzheimer’s disease [15]. All these neuronal disorders exhibit defects in the dendritic architecture of hippocampal pyramidal neurons and excitatory/inhibitory synaptic connectivity in the hippocampus, which are considered to play critical roles in cognitive function and dysfunction [16-18]. Hence, understanding the genetic influences on the effects of nicotine on cognition should contribute to the advancement of nicotine addiction treatment, although this area of research is relatively new.

However, not all individuals develop addiction to tobacco [19,20], nor the effects of nicotine on cognitive function are equal across human populations [21,22], suggesting that changes induced by nicotine consumption may be different in susceptible individuals. The *CHRNA5/A3/B4* gene cluster encoding the α5, α3 and β4 subunits of the nAChRs [23] is the major genomic locus associated with nicotine dependence in humans [24,25]. This region has also been linked to reduced levels of performance in cognitive domains such as response inhibition, attention and discriminative abilities [26,27]. Interestingly, Zhang et al. [28] demonstrated that current smoking corrects the increased perseverative errors and responses associated with this cluster in a cognitive flexibility test battery. These findings in human individuals support the view that some nicotine-dependent subjects may become addicted as a means of self-medication [29], explaining the fact that tobacco use is more prevalent and intense in human populations that manifest cognitive alterations [22].

We here tested the hypothesis that the *CHRNA5/A3/B4* cluster could modify the development of tobacco addiction and cognitive function by influencing neuroplasticity in the hippocampus, one of the few brain regions that expresses the α5, α3 and β4 nAChR subunits [30-36]. To this end, we used a BAC transgenic mouse model overexpressing the human *CHRNA5/A3/B4* gene cluster (Tg*CHRNA5/A3/B4*), which exhibit increased sensitivity to the pharmacological effects of nicotine [37], along with increased binding sites for nicotinic agonists in hippocampal membrane preparations [38] and hippocampal slices, particularly in the CA1 region [37]. We provide evidence that the overexpression of the *CHRNA5/A3/B4* region has an effect on the dendritic architecture of hippocampal pyramidal neurons, on the excitatory/inhibitory balance and on recognition memory. These structural and functional alterations in transgenic mice are rescued upon chronic nicotine administration.

## Materials and methods

### Animals

Transgenic mice overexpressing the human gene cluster *CHRNA5/A3/B4* (Tg*CHRNA5/A3/B4*) [37] were obtained from crosses of Tg*CHRNA5/A3/B4* males and B6/SJL-F1J females. Two transgenic Tg*CHRNA5/A3/B4* lines (L30 and L22; see our previous work [37]) were generated and showed no differences in their phenotype, excluding the possibility that the phenotypic profile of Tg*CHRNA5/A3/B4* and their response to nicotine might be attributed to the transgene insertion sites. In all experiments wild type (WT) littermates served as controls. Adult male mice (2–3 months of age) were group housed with 3–5 animals per cage under a 12 h light/dark schedule, in controlled environmental conditions of humidity (50–70%) and temperature (21 ± 1°C), with food and water supplied *ad libitum*. All experimental procedures were approved by the local ethical committee (CEEA - PRBB), and met the guidelines of the local (Catalan law 5/1995 and Decrees 214/97, 32/2007) the European regulations (EU directives 86/609 and 2001–486) and the Standards for Use of Laboratory Animals A5388-01 (NIH).

### Drug administration

(−)-Nicotine hydrogen tartrate was purchased from Sigma-Aldrich (St. Louis, MO). For the *in vivo* studies, animals were subcutaneously implanted with Alzet osmotic minipumps (Model 2001) (Alzet, Cupertio, CA) under O_2_ – isofluorane mixture anesthesia. Each minipump contained either saline (0.9% NaCl) or nicotine solutions (3.25 mg/Kg/d, free base) and delivered a constant subcutaneous flow in a rate of 1 μl/1 h. The concentration of nicotine was adjusted to compensate for body weight differences among subjects. This nicotine administration regime gives rise to sustained plasma nicotine concentrations similar to that reached in heavy smokers [39] and induces physical dependence in mice [40]. For the *in vitro* studies, (−) - nicotine was dissolved in fresh medium (3.25 μM, free base) and hippocampal primary cultures received medium alone or containing nicotine for 48 h.

### Histological analysis

All mice were deeply anesthetized with isofluorane and perfused with 0.1M phosphate buffer saline (PBS) followed by 4% paraformaldehyde (PFA, Sigma, St. Louis, MO). Mice were sacrificed on the seventh day after minipump implantation; the brains were removed and sliced at Bregma - 1.34 mm to - 2.18 mm.

### Morphometry of hippocampal pyramidal neurons

We used intracellular injections of *Lucifer yellow* (*LY*, L0259; Sigma, St. Louis, MO) [41,42]. Briefly, 150 μm thick vibratome-sliced sections were prelabeled with 4,6-diamidino-2-phenylindole (D9542; Sigma) and neurons from dorsal CA1 hippocampus were injected with *LY* by continuous current (n = 5–10 cells/animal; 4 – 5 animals/group from ≥ 3 experiments). After injecting the neurons, sections were first processed with anti-*LY* antibody made in rabbit (1:10 000, Sigma, L9163, in stock solution: 2% bovine serum albumin [A3425; Sigma], 1% Triton X-100 [30632; BDH Chemicals, Poole, UK], and 5% sucrose in PBS) and then with a biotinylated donkey anti-rabbit secondary antibody (1:200 in stock solution; RPN1004; Amersham Pharmacia Biotech, Little Chalfont, UK). Immunolabeled cells were visualized with Alexa Fluor 488-conjugated streptavidin (1:1000 in PBS, Invitrogen). Only cells identified as pyramidal neurons and whose entire apical dendrite arbor was completely filled were included in the analysis. Images were acquired at 40 x with a LSM 710 Zeiss confocal microscope equipped with laser excitation at 488 nm (Carl Zeiss MicroImaging GmbH, Germany) and each image was a *z* series projection of over 100 stacks, taken at 1 μm depth intervals. Individual neuronal apical arbors were three-dimensionally (3D) reconstructed using Neurolucida software (MicroBrightField, Inc., Vermont, USA) and morphometric analysis was performed using Neuroexplorer software (MicroBrightField, Inc., Vermont, USA) [43]. This module opens an image stack of confocal images and allows the measurement of several morphological parameters of dendritic arborisation. The following parameters were analysed: Sholl analysis (branching complexity, measured as the number of dendritic intersections within concentric 10 μm radial spheres, calculated as a function of distance from the soma), total number of nodes, total dendritic length, dendritic volume (the volume of a neuron’s apical dendritic field calculated as the volume enclosed by a polygon created by joining the most distal points of the dendritic processes, the 3D convex Hull volume) [44].

To further investigate neuronal structure of pyramidal neurons, Tg*CHRNA5/A3/B4* males were crossed with *Thy1-YFP (Yellow Fluorescent Protein)* heterozygous females (B6.Cg-Tg (*Thy1-YFPH*) 2Jrs/J; Jackson Laboratories) and obtained double transgenic mice that expressed *YFP* sparsely in subsets of pyramidal neurons. Brains were extracted as described before, postfixed with 4% PFA and cryoprotected with 30% sucrose. A vibratome (Leica, Wetzlar, Germany) was used to obtain coronal sections (150 μm thick). Fluorescent images were obtained with a SP5 confocal microscope (Leica, Wetzlar, Germany) and analyzed by ImageJ software. The number of basilar (*stratum oriens*) and apical (*stratum radiatum*) dendritic structures of CA1 pyramidal neurons was determined within a 50 × 50 μm^2^ area in a single plane images acquired at 40 × (n = ≥100 images animal; 4–5 animals/group from ≥ 3 experiments). The dendritic spine density of CA1 pyramidal neurons was quantified along 30 μm length sections of primary and secondary dendrites, at 50 μm from the cell soma and, expressed as number of spines per 10 μm (n = 5–10 cells/animal; 4–5 animals/group from ≥ 3 experiments). Spine count was performed on images acquired at 63 × with 2.0 × (optical zoom), generating a stack from 9 – 10 images, taken at 0.36 μm depth intervals. Dendritic spines were separated on morphological categories as previously described (stubby, mushroom-like, thin or filopodia) [45]. The number of pyramidal cells per CA1 and CA3 areas was quantified on images acquired at 20 × and taken at different *x*, *y* and *z* coordinates to visualize the entire hippocampus. Each image was a *z* series projection of approximately 10–12 stacks, and taken at 5 μm depth intervals.

### Immunohistochemistry

Vesicular glutamate transporter (VGLUT1) and vesicular GABA transporter (VGAT) were used as markers for excitatory and inhibitory synaptic inputs [46]. 40 μm thick coronal sections were obtained from brains extracted as described before, using a cryostat (Leica, Wetzlar, Germany). Samples were permeabilized with 0.1M PBS 0.5% Triton X-100 (PBST) (Sigma, St. Louis, MO), and blocked in 20% fetal bovine serum (FBS) PBST, for 1 h at room temperature (RT). Samples were incubated with the primary antibodies anti-VGLUT1 (mouse, 1:200, Synaptic Systems, Goettingen, Germany) and anti-VGAT (guinea pig, 1:200, Synaptic Systems, Goettingen, Germany), in 5% FBS PBST, overnight at 4°C. After washes in PBST, they were incubated with secondary fluorescently labeled antibodies (Alexa ®Fluor 488 and Alexa ®Fluor 594, Life Technologies, Grand Island, NY) in 5% FBS PBST for 1 h, at RT. Images were acquired with SPE Confocal Microscope (Leica, Wetzlar, Germany), connected to the LAS AF software (Leica, Wetzlar, Germany), and analyzed using Image J software. Background was subtracted from negative control values of each sample and the same threshold was applied for each channel. The number of VGLUT1 and VGAT puncta was quantified within a 20 × 20 μm^2^ region of the CA1 *strata oriens* and *radiatum* on single plane images obtained at 63 × with 5.0 × (optical zoom) (n = ≥100 images animal; 4–5 animals/group from ≥ 3 experiments).

### Electrophysiological recordings

Field excitatory postsynaptic potentials (fEPSPs) were recorded in the *stratum radiatum* of dorsal hippocampal CA1 region in response to stimulation of the Schaffer collateral (SC) pathway (n = 3–5 recordings/animal; 4 – 5 animals/group from ≥ 3 experiments). Mice were sacrificed by decapitation on the seventh day after minipump implantation, the brain was quickly removed and placed on ice-cold cutting solution (in mM): 2.5 KCl; 3 MgSO_4_; 1.25 NaHPO_4_; 1 CaCl_2_; 26 NaHCO_3_; 10 sucrose and aerated with 95% O_2_ – 5% CO_2_ to a final pH of 7.4. Coronal slices (400 μm thick) were obtained with a vibratome (Leica, Wetzlar, Germany); placed in an interface style recording chamber (Fine Science Tools, Foster City, CA) and bathed in artificial cerebrospinal fluid (ACSF) containing (in mM): 124 NaCl; 2.5 KCl; 1 MgSO_4_; 1.25 NaHPO_4_; 2.5 CaCl_2_; 26 NaHCO_3_; and 10 dextrose aerated with 95% O_2_–5% CO_2_ to a final pH of 7.4. Bath temperature was maintained at 32 – 34°C. Unfiltered recordings were obtained by means of ACSF-filled glass electrodes (impedance 1–2 MΩ) through a Neurolog system (Digitimer) amplifier. Electrical stimuli were delivered using a stimulus isolator unit in constant current mode (WPI, Sarasota, FL) with a concentric monophasic bipolar electrode (200-μm-diameter ultra-small concentric bipolar electrode; Frederick Haer Co., Bowdoinham, ME). Stimulus strength was adjusted to a stimulation intensity that yielded a half-maximal response (50–150 μA). For each slice, after establishing a stable response (electrical stimulation at 0.03 Hz), paired-pulse facilitation (PPF) was induced by a double-pulse (50 ms apart) stimulation protocol. PPF is a short-term presynaptic phenomenon that at SC-CA1 synapses it is inversely related to the probability of neurotransmitter release [47]. Long-term potentiation (LTP) was induced by a high frequency stimulation protocol (HFS; 100 Hz; 1 s) 20 min after baseline recording. Responses were recorded for a period of 60 min (pulse at 0.03 Hz) and the magnitude of LTP was measured by averaging the percent increase of the fEPSP slope compared with baseline. Recordings were acquired, digitized, and analyzed using a data acquisition interface and software from Cambridge Electronic Design (Spike2).

### Cell culture

Hippocampal primary cultures were obtained from WT and Tg*CHRNA5/A3/B4* mice at embryonic day 17.5 - 18.5 (n = 4 independent experiments, 3–4 cultures/condition from ≥ 3 experiments). Cultures were obtained from individual embryos; no pool of material was mixed. Hippocampi were dissected on ice-cold Dulbecco’s Modified Eagle Medium (DMEM) (Life Technologies, Grand Island, NY). Cells were mechanically dissociated, centrifuged and resuspended in 0.5 ml Neurobasal culture medium, supplemented with 2% B27 factor, 1% Glutamax, 0.5% Penicillin/Streptomycin (NB^+++^) and 10% Inactivated Horse Serum (all reagents were supplied from Life Technologies, Grand Island, NY). Neurons were seed at a density of 5 × 10^4^ cells per well on round glass coverslips and, incubated under culture conditions of 37°C and 5 %CO_2_. 24 h after plating, the serum was removed and substituted by NB^+++^ to avoid massive glial proliferation. To analyze the morphology of pyramidal neurons, at *day-in-vitro* (*DIV*) 5, cultures were transfected with a plasmid containing enhanced green fluorescent protein (*EGFP*) driven by the *Thy1* promoter (designed and kindly provided by Dr. G. Ramakers, University of Amsterdam), by means of Lipofectamine 2000 (Life Technologies, Grand Island, NY) and following manufacturer instructions. At *DIV*7, half of the plate received fresh medium alone or containing nicotine for 48h. All the morphological and biochemical studies were performed in *DIV*9 cultures fixed in PFA 4% for 20 min at RT. The dendritic complexity of pyramidal neurons was examined in non-overlapping positive cells expressing *EGFP*, by means of Sholl analysis (n = 4–5 cells/culture, 3–4 cultures/condition from ≥ 3 experiments). Images were acquired with a SP5 Confocal Microscope (Leica, Wetzlar, Germany), connected to the LAS AF software (Leica, Wetzlar, Germany), using a 63x objective generating a *z* - stack from 7–9 images taken at a 0.5 μm depth interval, and at different *x*, *y* and *z* coordinates to visualize the entire neuron. For spine count on transfected neurons, images were acquired at 63 × with 2.0× (optical zoom), generating a stack from 9–10 images taken at a 0.36 μm depth interval. Spines were quantified along 30 μm length sections of primary and secondary dendrites at a distance of 50 μm from the cell soma, and expressed as number of spines per 10 μm of dendrite length (n = 4 – 5 cells/culture, 3–4 cultures/condition from ≥ 3 experiments). To examine glutamatergic and GABAergic inputs in the neuronal cultures, immunofluorescence labeling against VGLUT1 and VGAT markers was performed as describe above. The number of VGLUT1 and VGAT puncta per 10 μm of dendrite was determined within a region of 50 μm proximal to the cell body of neurons, with aids of Image J software (n = 4 – 5 cells/culture, 3 – 4 cultures/condition from ≥ 3 experiments). To assess the viability of the cultures at *DIV9*, neurons were incubated with 3-(4,5-dimethyolthiazol-2-yl)-2,5-diphenyltetrazlium bromide (MTT, 500 μg/ml, Sigma, St. Louis, MO) in 0.1M PBS for 30 min, at 37°C and, under dark conditions. After incubation, the medium was removed and formazan dye was extracted using 100% detergent sodium dodecyl sulfate (DMSO, Sigma, St. Louis, MO). The absorbance was determined using a microplate reader at 550 nm (n = 4–5 wells/culture, 3–4 cultures condition from ≥ 3 experiments).

### Behavioral analysis

Adult male mice were allowed to habituate to the testing room under dim light for at least 30 min. Animals (n = 10–12 mice/group from ≥ 3 experiments) were subjected for novel object recognition memory paradigm on the fourth day after minipumps were implanted. Mice that underwent behavioral tests were never used for histological or electrophysiological analysis.

### Novel object recognition (NOR)

The apparatus consisted of a rectangular open-field arena (70 cm long × 70 cm wide × 30 cm high) made of Plexiglas, surrounded by curtains to avoid the influence of external stimuli during the experiment. Animals’ behavior was monitored using System Motor Activity Record and Tracking software (SMART, Panlab Harvard Apparatus, Spain). On the first day, mice were habituated to the arena for 10 min. On the second day, mice were presented with two identical objects, for 10 min. Subjects failing to complete a minimum of 20 s of exploration during the familiarization session were excluded for posterior analysis. In a 1 h delay (test session), mice were presented with one familiar object and a novel one, for 5 min. The discrimination index was calculated as time exploring the novel object – time exploring the familiar object/total time of exploration *100 [48]. Exploratory behavior was defined as the animal directing its nose towards the object at a distance of < 2 cm and manually registered by the experimenter. Sitting on or resting against the object was not considered as exploration. All the objects used were plastic made and induced similar exploration levels. The arena and objects were deeply cleaned between animals to avoid olfactory cues. Anxiety-like behavior was measured as percentage of time spent in the periphery of the open field.

### Statistical analyses

All data are presented as mean ± standard error of mean (SEM). Genotype comparison was performed by Student’s T test. Two-way analysis of variance (ANOVA) was used for genotype and treatment analysis. When the interaction of genotype x treatment was significant a Bonferroni *post hoc* test was used as a correction between pair wise comparisons. Otherwise, the significant effect of genotype or treatment was taken into consideration. Repeated measure ANOVA was used for the Sholl analysis. All statistical analyses were performed using Statistical Package for the Social Sciences (SPSS) software (version 19.0).

## Results

### *CHRNA5/A3/B4* overexpression leads to a reduced dendritic complexity in CA1 hippocampal pyramidal neurons that is rescued by nicotine treatment

**Figure 1:**
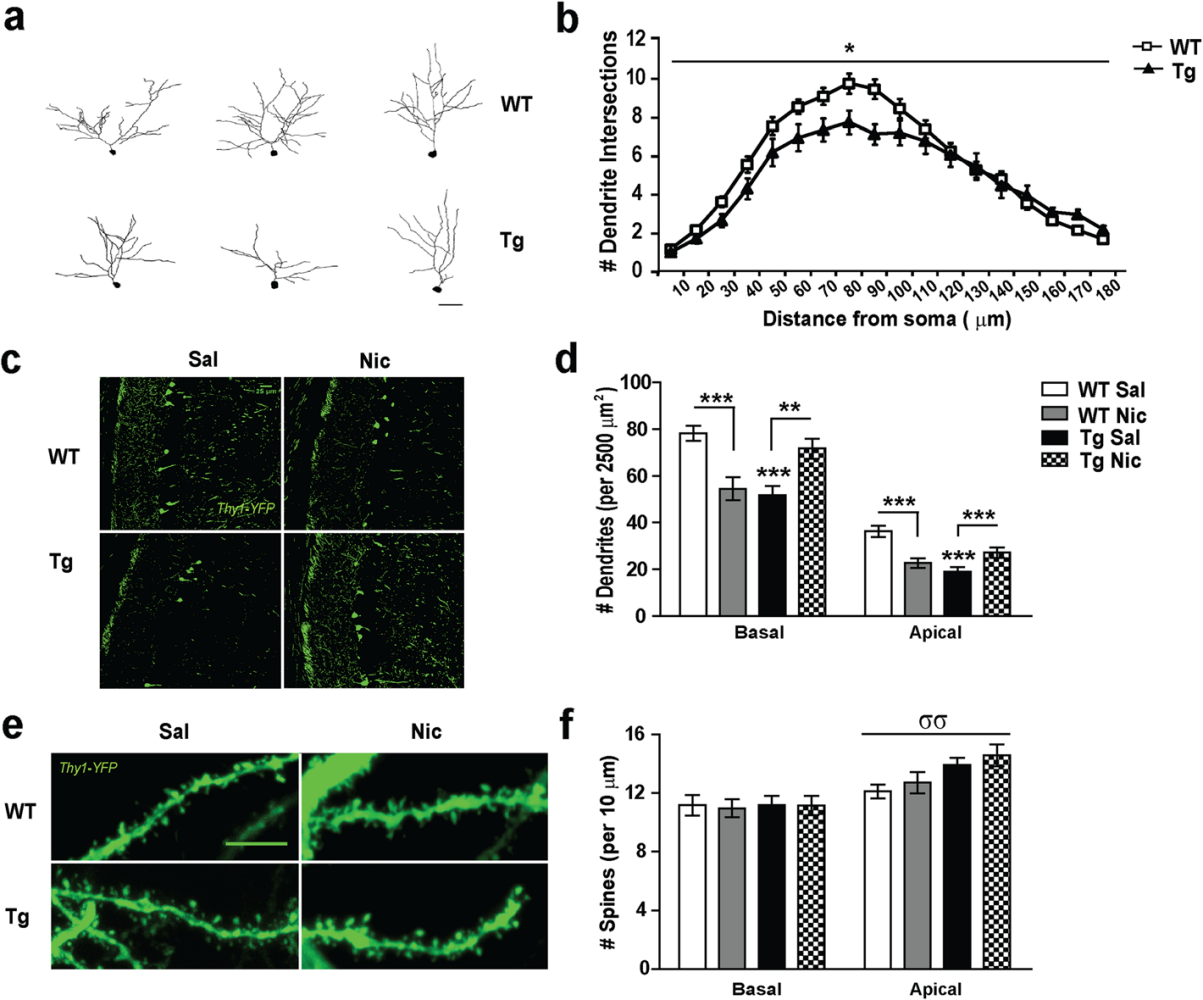
**CA1 pyramidal neurons structure in mice overexpressing the** ***CHRNA5/A3/B4*** **region and upon chronic nicotine treatment.** **(a)** Representative reconstructions of the apical dendritic tree of *Lucifer Yellow* transfected wild type (WT, upper panel) and Tg*CHRNA5/A3/B4* neurons (Tg, lower panel), Scale bar 25 μm. **(b)** Sholl analysis indicated that transgenic neurons showed reduced number of dendritic intersections compared to WT, particularly at 50 to 150 μm from the cell soma (n = 5–10 cells/animal; 4–5 animals/group from ≥ 3 experiments). **(c)** Representative confocal images of the CA1 region in *Thy1-Yellow Fluorescent Protein (YFP) -* WT and Tg mice that received either saline (Sal) or nicotine (Nic, 3.25 mg/Kg/d) for 7 d, Scale bar 25 μm. **(d)** Quantification of the number of apical and basal dendritic structures in 50 × 50 μm^2^ area revealed significant reductions in Sal-treated *Thy1-YFP-*Tg mice, compared to Sal-treated *Thy1-YFP-*WT mice. Chronic administration of nicotine restored the dendritic deficit in *Thy1-YFP-*Tg mice but instead the same treatment reduced the number of dendritic structures in *Thy1-YFP-*WT mice (n = ≥100 images animal; 4–5 animals/group from ≥ 3 experiments). **(e)** Representative photomicrograph illustrating dendritic spines on apical dendrites in CA1 pyramidal neurons from saline and nicotine treated *Thy1-YFP-* WT and Tg mice, Scale bar 10 μm. **(f)** Quantification of spines per 10 μm of dendrite length indicated that apical but not basal dendrites from saline- and nicotine-treated *Thy1-YFP-*Tg mice presented increased spines as compared to *Thy1-YFP-*WT (n = 5–10 cells/animal; 4–5 animals/group from ≥ 3 experiments). *p ≤ 0.05, **p ≤ 0.01, ***p ≤ 0.001; Two-way ANOVA genotype effect σσ *p* ≤ 0.01.

Using *Lucifer Yellow (LY)* injections and neuronal reconstruction techniques, we detected a reduction in the complexity of the apical dendritic tree in Tg*CHRNA5/A3/B4* CA1 pyramidal neurons, compared to WT (*p* = 0.019, Figure 1a-b, Sholl analysis), with decreased number of nodes (*p* < 0.001, Additional file 1: Figure S1a). This decrease of branching complexity in transgenic neurons was found only in the proximal region of the apical dendrite, which corresponds to the portion of dendrite mainly receiving excitatory input from CA3 connections, but not in the distal part receiving input from the temporoammonic pathway [49]. Transgenic CA1 pyramidal neurons presented reduced total dendrite length (*p* = 0.014) and total dendrite volume (*p* = 0.016, Additional file 1: Figure S1b-c).

We next investigated if a chronic nicotine treatment in the adult brain affects the dendritic complexity of CA1 pyramidal neurons. To address this question, we used transgenic animals expressing YFP in pyramidal neurons (*Thy1-YFP*-WT and *Thy1-YFP*-Tg*CHRNA5/A3/B4* mice) implanted with saline or nicotine (3.25 mg/Kg/d) for 7 d. In saline-treated *Thy1-YFP* Tg*CHRNA5/A3/B4* mice we again observed a significant reduction in the number of proximal apical dendrites (*p* < 0.001) and in the proximal portions of basal dendrites (*p* < 0.001) (Figure 1c-d). Chronic nicotine treatment in *Thy-YFP-*Tg*CHRNA5/A3/B4* mice rescued this phenotype and increased the number of basal (*p* = 0.007) and apical dendrites (*p* < 0.001) (Figure 1c-d), but reduced both the number of basal (*p* < 0.001) and apical dendrites (*p* < 0.001) in *Thy-YFP-*WT mice (Figure 1c-d). No differences in the number of pyramidal cell somas per area in the CA1 *stratum pyramidale* layer were detected between genotypes or treatment groups (Additional file 2: Figure S2a).

### Dendritic spine density in CA1 pyramidal neurons increases in WT and Tg*CHRNA5/A3/B4* mice upon chronic nicotine treatment

*Thy1-YFP*-WT and Tg*CHRNA5/A3/B4* mice treated with either saline or nicotine (3.25 mg/Kg/d for 7 d) were used to examine whether the altered dendritic branching in CA1 pyramidal neurons was accompanied with changes in dendritic spines. Basal dendrites from transgenic neurons did not show differences in total dendritic spines as compared to WT (Figure 1e-f). Nevertheless, they presented an increase in the proportion of stubby spines (*p* < 0.001) along with a non-significant reduction of thin spines (Table 1). In contrast, apical dendrites of *Thy1-YFP*-Tg*CHRNA5/A3/B4* pyramidal neurons presented increased density of total dendritic spines, as compared to WT (*p* < 0.001, Figure 1e-f). Morphological analysis of spines indicated that, similar to the spines of basal dendrites, this increase was mainly due to increased density of stubby spines (*p* = 0.039) but also of mushroom-like (*p* = 0.01) (Table 1).

**Table 1:** **Effects of nicotine on dendritic spines in CA1 pyramidal neurons from adult mice**

	**Apical dendrites**			**Basal dendrites**		
**Spines per 10 μm**	**Stubby**	**Mushroom-like**	**Thin**	**Stubby**	**Mushroom-like**	**Thin**
WT Sal	0.90±0.12	10.94±0.45	0.27±0.08	0.84±0.09	10.30±0.65	0.03±0.02
WT Nic	0.79±0.17	11.83±0.65^ω^	0.08±0.06	0.86±0.12	10.10±0.58	0.00±0.00
Tg Sal	1.50±0.23^σ^	12.20±0.39^σ^	0.23±0.10	1.16±0.11^σ^	10.01±0.62	0.01±0.01
Tg Nic	0.96±0.15^σ^	13.51±0.70^σ,ω^	0.11±0.06	1.40±0.13^σ^	9.74±0.61	0.00±0.00

Number of spines per 10 μm of dendrite length, in apical and basal dendrites of CA1 pyramidal neurons from *Thy1-YFP-*wild type (WT) and *Thy1-YFP-*Tg*CHRNA5/A3/B4* (Tg). Mice were implanted with osmotic minipumps delivering either saline (Sal) or nicotine (Nic, 3.25 mg/Kg/d) for 7 d (n = 5–10 cells/animal; 4–5 animals/group from ≥ 3 experiments). Two-way ANOVA genotype effect ^σ^ *p* ≤ 0.05, treatment effect ^ω^ *p* ≤ 0.05.

Chronic nicotine treatment increased the density of mushroom-like spines on apical dendrites (*p* = 0.05, Table 1), without changing the total dendritic spine density, in both *Thy1-YFP*-WT and *Thy1-YFP*-Tg*CHRNA5/A3/B4* mice (Figure 1e-f**)**. However, nicotine did not affect spine densities in CA1 pyramidal basal dendritic tree.

### Reduced excitatory and inhibitory synaptic inputs in the CA1 region of Tg*CHRNA5/A3/B4* mice are normalized upon chronic nicotine administration

In the mature brain, most glutamatergic synapses occur at dendritic spines [45,50]. Thus, the differences observed in dendritic spines might be associated with changes in the density of excitatory synaptic inputs in the CA1 region. To explore this possibility, we immunostained hippocampal slices from saline or nicotine treated WT and Tg*CHRNA5/A3/B4* mice using VGLUT1, a commonly used neuronal marker for presynaptic glutamatergic synapses in the mouse hippocampus [51].

**Figure 2:**
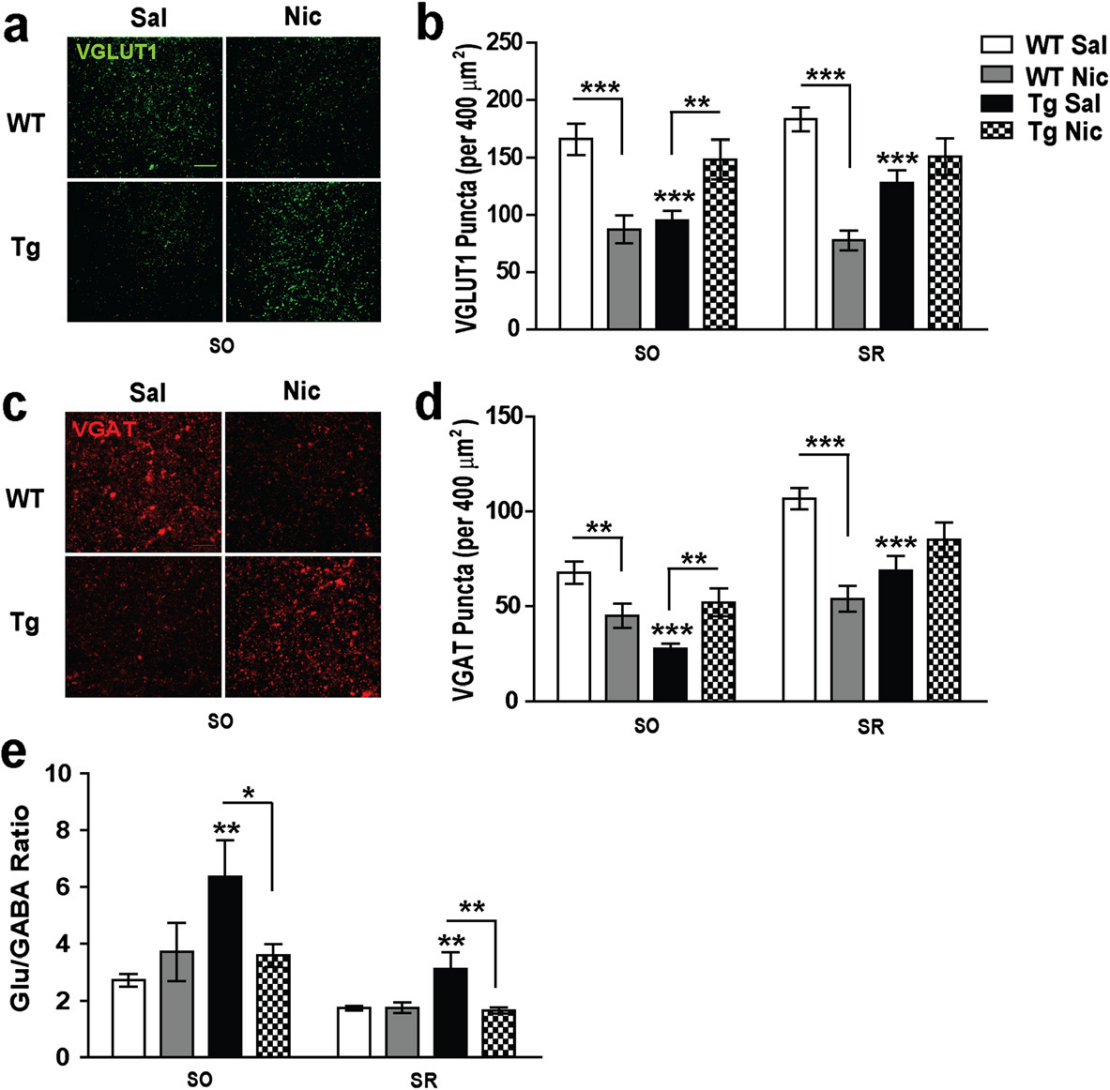
**Glutamatergic and GABAergic puncta and their ratio in the hippocampal CA1 region.** **(a)** Representative confocal images of VGLUT1 immunostaining in the *stratum oriens* (SO) of wild type (WT) and Tg*CHRNA5/A3/B4* (Tg) mice that received either saline (Sal) or nicotine (Nic, 3.25 mg/Kg/d) for 7 d, Scale bar 5 μm. **(b)** Quantification of VGLUT1 puncta showing that Tg mice presented reduced glutamatergic puncta in SO and stratum *radiatum* (SR), as compared to their WT littermates. Chronic infusion of nicotine rescued the reduced density of VGLUT1 puncta in the CA1 SO of Tg mice, but caused opposite effects in WT, leading to a significant decrease both in SO and SR. **(c)** Representative confocal images of VGAT immunostaining in the SO of mice with the conditions indicated, Scale bar 5 μm. **(d)** Quantification of VGAT puncta showing reduced GABAergic input in SO and SR of Tg mice, compared to WT. Chronic nicotine increased VGAT puncta in SO in Tg. In contrast, the same treatment reduced VGAT puncta in SO and SR in WT mice. **(e)** The glutamatergic (Glu)/GABAergic ratio in CA1 SO and SR regions was increased in Tg mice, as compared to WT. Chronic nicotine treatment rescued the increased Glut/GABA ratio in Tg, with no effect in WT (n = ≥100 images animal; 4–5 animals/group from ≥ 3 experiments). *p ≤ 0.05, **p ≤ 0.01, ***p ≤ 0.001.

Tg*CHRNA5/A3/B4* exhibited decreased VGLUT1 puncta in *strata radiatum* (*p* < 0.001) and *oriens* (*p* < 0.001) (Figure 2a-b) that was restored to control values upon chronic nicotine administration (3.25 mg/Kg/d for 7 d), being the restoration more pronounced in *stratum oriens* (*p* = 0.003) than *stratum radiatum* (Figure 2a-b). In contrast, the same treatment had opposite effects in WT animals, leading to a loss of glutamatergic inputs in the CA1 region (*stratum radiatum p* < 0.001; *stratum oriens p* < 0.001, Figure 2a-b).

When examining the density of inhibitory synapses (VGAT puncta), we found similar results, since Tg*CHRNA5/A3/B4* showed significantly reduced GABAergic inputs (*stratum radiatum p* < 0.001, *stratum oriens p* < 0.001, Figure 2c-d). However, the reduction of VGAT exceeded the reductions of VGLUT1 puncta and thus, the ratio of glutamatergic vs. GABAergic input was increased in the CA1 region of Tg*CHRNA5/A3/B4* mice, causing an E/I imbalance (*stratum radiatum p* = 0.004, *stratum oriens p* = 0.007, Figure 2e). In all groups of mice the density of GABAergic input was higher in *stratum radiatum* than in *stratum oriens* (Figure 2c), reflecting a greater inhibitory regulation in this region, as previously described [52]. Chronic nicotine normalized the density of GABAergic inputs in Tg*CHRNA5/A3/B4* (*p* = 0.002, Figure 2c-d) and compensated for their E/I imbalance (*stratum radiatum p* = 0.003; *stratum oriens p* = 0.039, Figure 2e). Conversely, chronic nicotine reduced GABAergic inputs in WT mice (*stratum radiatum p* < 0.001; *stratum oriens p* = 0.006, Figure 2c-d), but without modifying their E/I balance (Figure 2e). The treatment did not modify the density of CA3 pyramidal neurons neither in WT nor in Tg*CHRNA5/A3/B4* mice, the major excitatory inputs in the CA1 region (Additional file 2: Figure S2b).

### Chronic nicotine enhances HFS-induced LTP at SC-CA1 connections in WT and Tg*CHRNA5/A3/B4* mice

**Figure 3:**
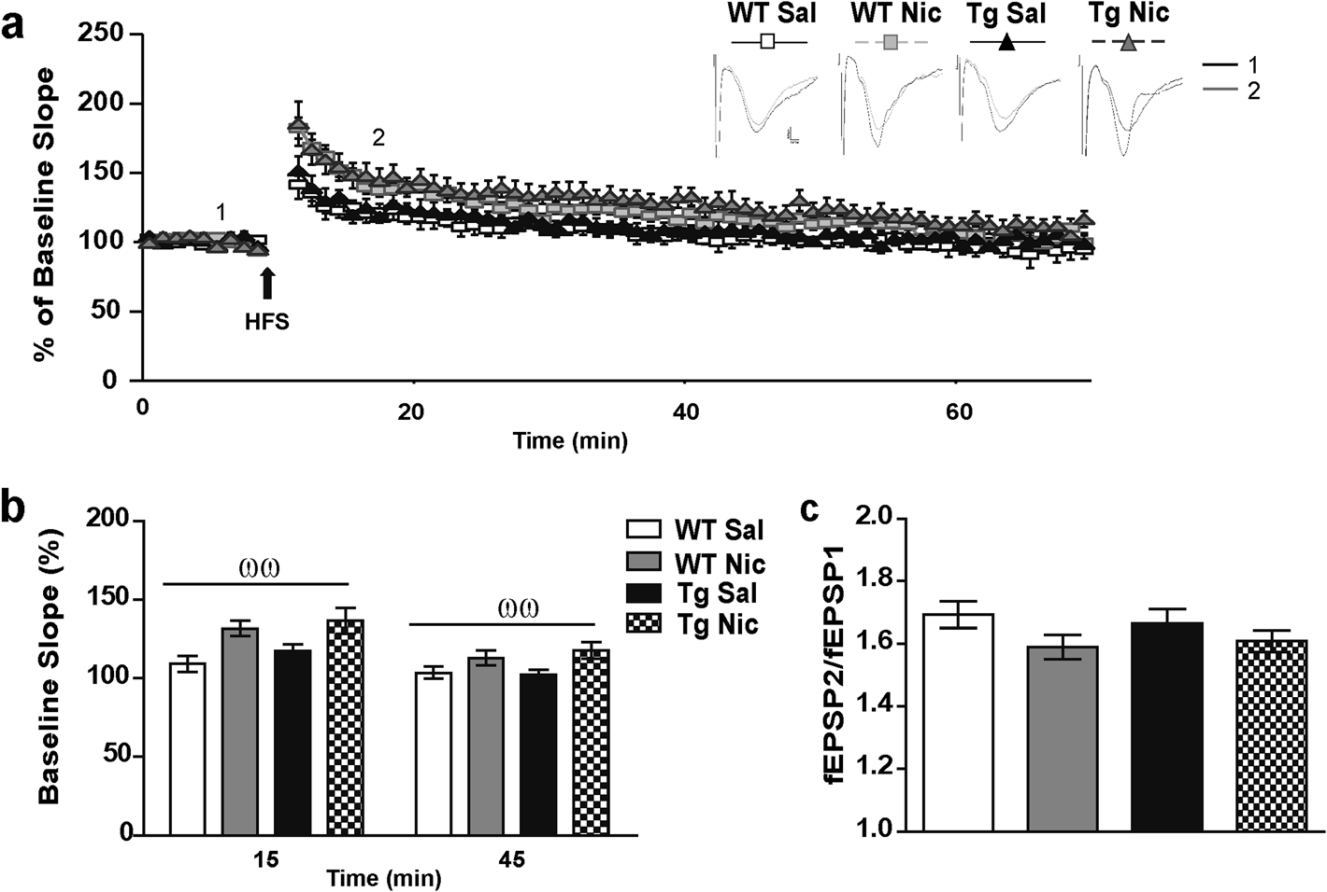
**Electrically induced synaptic plasticity of Shaffer collateral-CA1 pathway.** **(a)** High frequency stimulation (HFS; 100Hz, 1s) induced synaptic potentiation at Schaffer Collateral (SC)-CA1 synapses similarly in slices from saline (Sal)-treated wild type (WT) and Tg*CHRNA5/A3/B4* (Tg) mice. Chronic nicotine (Nic, 3.25 mg/Kg/d) administration for 7 d enhanced synaptic potentiation both in WT and Tg mice. Upper panel: representative field excitatory postsynaptic potentials (fEPSPs) in response to one stimulation in the CA1 *stratum radiatum* before (1) and 15 min after (2) applying HFS (arrow), Scale bar 0.5 mV and 1 ms. Lower panel: slopes of the fEPSPs normalized to those obtained before applying HFS (%). **(b)** Mean values of fEPSPs potentiation at 15 and 45 min after HFS. **(c)** The paired pulse ratio (fEPSP2/fEPSP1) with an inter-pulse interval of 50 ms (response average of 5 paired pulses; time interval 1 s) was similar among slices from the different experimental conditions (n = 3–5 recordings/animal; 4 – 5 animals/group from ≥ 3 experiments). Two-way ANOVA treatment effect ωω *p* ≤ 0.01.

We further interrogated whether differences in excitatory and inhibitory synapses described above could underlie changes in synaptic strength, such as LTP responses, considered the cellular substrate of memory function [53]. To this aim, we recorded fEPSPs in the CA1 *stratum radiatum* region evoked by HFS (100 Hz, 1 s) applied to SC afferents. Recordings were performed in slices from WT and Tg*CHRNA5/A3/B4* animals treated with saline or chronic nicotine (3.25 mg/Kg/d for 7 d). The results showed that HFS stimulation to SC amplified the degree of CA1 synaptic potentiation along the 60 min of recordings, similarly in slices from saline-treated Tg*CHRNA5/A3/B4* and WT mice (Figure 3a-b). Additionally, using paired pulse facilitation (50 ms apart) no differences were observed between genotypes in release probability (Figure 3c).

Chronic nicotine administration reduced the threshold for LTP induction at SC-CA1 pathway (Figure 3a-b). Slices from mice that received the drug treatment elicited increased synaptic potentiation immediately upon stimulation (at 15 min post HFS, *p* = 0.002, Figure 3b) that was maintained for more than 45 min (*p* = 0.009, Figure 3b). Noticeably, the effects of nicotine on LTP induction were similar between genotypes. Moreover, chronic nicotine had no significant effect on paired pulse facilitation (Figure 3c).

### Reduced dendrite arborization of cultured Tg*CHRNA5/A3/B4* hippocampal neurons is rescued by nicotine

The morphological features of pyramidal neurons in the brain are influenced by the anatomical connections with other structures. To dissociate these histological effects we used primary cultures and investigated the structure of hippocampal pyramidal neurons in Tg*CHRNA5/A3/B4* and their response to nicotine in more controlled conditions. *Thy1-EGFP* transfected hippocampal primary cultures from WT and Tg*CHRNA5/A3/B4* embryos were exposed for 48h to basal medium (control) or medium with nicotine (3.25 μΜ).

**Figure 4:**
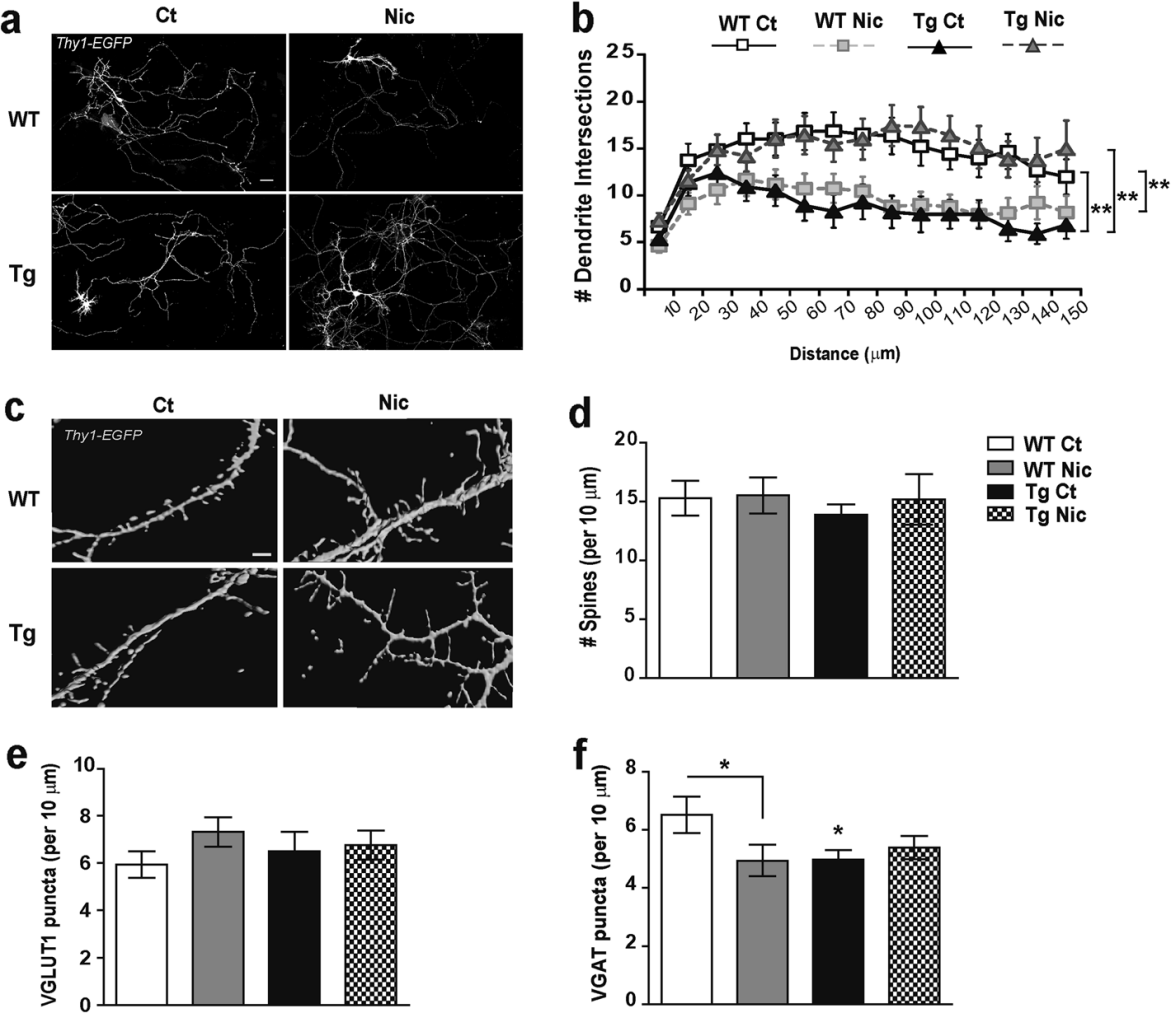
**Pyramidal neurons structure in hippocampal primary cultures.** **(a)** Photomicrograph illustrating positive *Thy1-Enhanced Green Fluorescent Protein (EGFP)* pyramidal neurons from wild type (WT) and Tg*CHRNA5/A3/B4* (Tg) cultures exposed to medium alone (Control, Ct) or containing nicotine (Nic, 3.25 μM) for 48 h, Scale bar 25 μm. **(b)** Sholl analysis revealed reduced number dendritic complexity in neurons from Tg cultures, as compared to WT. Chronic exposure to nicotine rescued the reduced dendritic complexity in Tg cultures. In contrast, in WT cultures the same treatment reduced the dendritic arborisation. **(c)** Photomicrograph illustrating dendritic spines in *EGFP* transfected neurons from the experimental conditions indicated, Scale bar 2 μm. **(d)** Quantification of spines per 10 μm of dendrite length showed no differences among groups. Quantification of the number of **(e)** VGLUT1 and **(f)** VGAT puncta per 10 μm of dendrite length in cultures from the experimental conditions indicated. No differences in VGLUT1 puncta were detected among groups. Instead, VGAT puncta were reduced in Tg, as compared to WT cultures. Nicotine treatment reduced VGAT inputs only in WT but not in Tg cultures (n = 4–5 cells/culture; 3 – 4 cultures/condition from ≥ 3 experiments). *p ≤ 0.05, **p ≤ 0.01.

Similar to the adult mouse tissue, the dendritic complexity of cultured hippocampal pyramidal neurons from transgenic embryos was significantly reduced compared to WT cultures (*p* = 0.004, Figure 4a-b, Sholl analysis). In transgenic pyramidal neurons, we also detected increased density of immature filopodia-like structures compared to WT neurons (*p* = 0.007), while no significant changes were detected in other spines subtypes (Table 2) or the density of total dendritic spines (Figure 4c-d). Cultures from transgenic embryos also presented a significant reduction in GABAergic inputs (VGAT puncta) (*p* = 0.025, Figure 4f), but no changes in glutamatergic input (VGLUT1 puncta) (Figure 4e), confirming that overexpression of the *CHRNA5/A3/B4* cluster causes a significant decrease of GABAergic input.

**Table 2:** **Effects of nicotine exposure on dendritic spines in pyramidal neurons from hippocampal primary cultures**

**Spines per 10 μm**	**Filopodia**	**Stubby**	**Mushroom-like**	**Thin**
WT Ct	0.56±0.15	2.23±0.72	12.05±1.44	1.00±0.24
WT Nic	0.93±0.22	0.94±0.29^ω^	12.82±1.32	1.75±0.37
Tg Ct	1.61±0.35^σ^	1.18±0.34	10.96±0.86	1.72±0.31
Tg Nic	2.29±0.68^σ^	0.65±0.23^ω^	12.21±2.21	2.32±0.44

Number of spines per 10 μm of dendrite length, in neurons from wild type (WT) and Tg*CHRNA5/A3/B4* (Tg) embryos. Cultures were incubated with medium alone (Ct, Control) or containing nicotine (Nic, 3.25 μM) for 48 h (n = 4–5 cells/culture; 3 – 4 cultures/condition from ≥ 3 experiments). Two-way ANOVA genotype effect σ *p* ≤ 0.05, treatment effect ω *p* ≤ 0.05.

Again, nicotine differentially affected the dendritic complexity and presynaptic inputs depending on the genotype. Sholl analysis revealed that while nicotine was able to restore the dendritic complexity in transgenic neurons (*p* = 0.002), the same treatment caused detrimental effects on WT cultures reducing the dendritic arborization (*p* = 0.009, Figure 4a-b, Sholl analysis). Nicotine exposure did not modify the density of glutamatergic inputs neither in WT or transgenic cultures (Figure 4e), but reduced stubby spines (*p* = 0.033), without affecting filopodia and mushroom-like spines (Table 2) or the total spine density (Figure 4c-d). However, nicotine reduced VGAT puncta in control cultures (*p* = 0.025), while it had no effect in transgenic cultures (Figure 4f). The MTT control assay revealed no significant differences among groups (WT control 1.00 ± 0.00; WT nicotine 0.84 ± 0.123; Tg*CHRNA5/A3/B4* control 1.39 ± 0.297; Tg*CHRNA5/A3/B4* nicotine 1.24 ± 0.251), indicating that the viability of the culture was not affected by the nicotine exposure.

### Impaired novelty recognition memory in Tg*CHRNA5/A3/B4* mice is rescued upon chronic nicotine treatment

**Figure 5:**
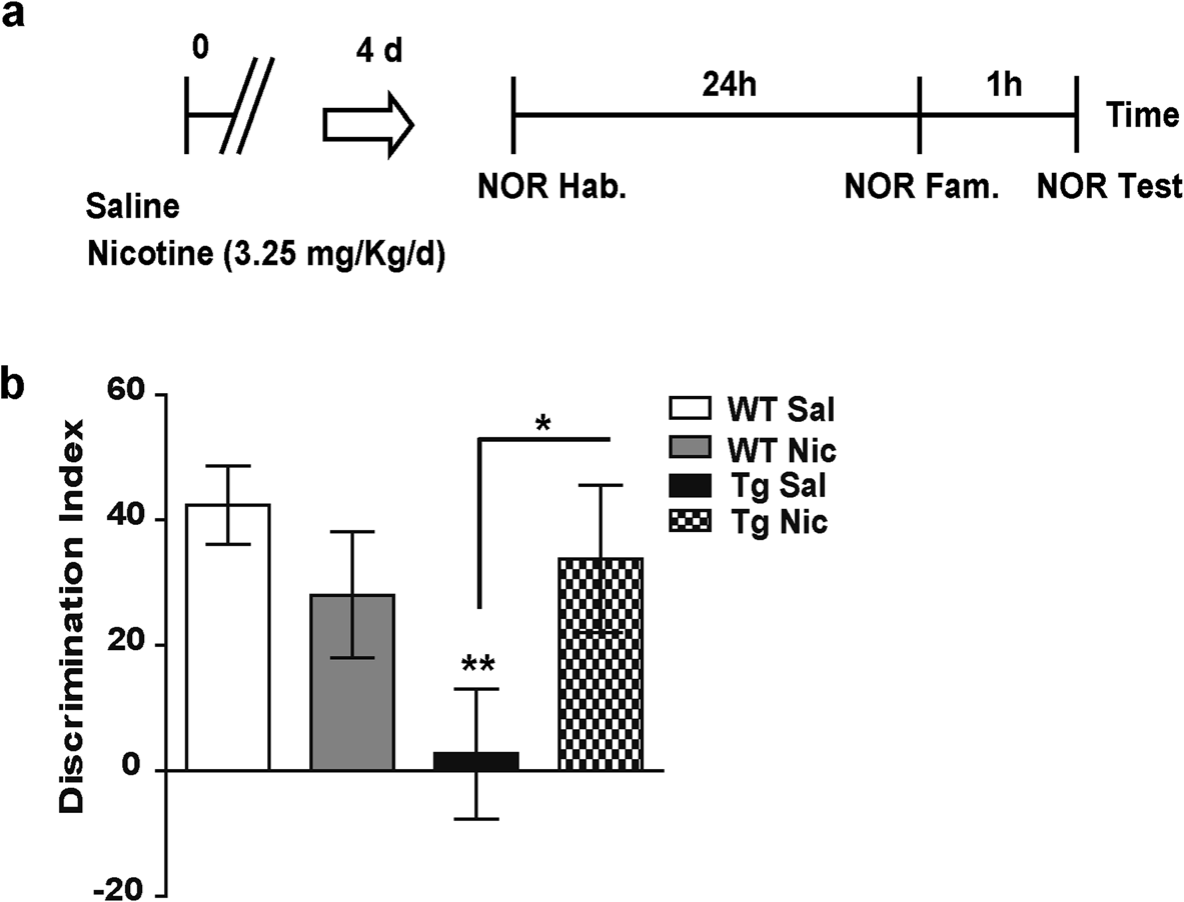
**Short-term novel object recognition memory.** **(a)** Drug administration protocol used during the novel object recognition (NOR) task (Hab, Habituation session; Fam, Familiarization session; Test session). **(b)** Saline (Sal)-treated Tg*CHRNA5/A3/B4* (Tg) mice presented a significant impairment in recognition memory, as compared to wild type (WT) littermates, as shown by reduced discrimination index which was expressed as [(time exploring the novel object – time exploring the familiar object)/total time of exploration*100]. Chronic administration of nicotine (Nic, 3.25 mg/kg/d) for 5 d rescued the impaired recognition memory in Tg mice, but had no effect in WT (n = 10–12 animals/group from ≥ 3 experiments). *p ≤ 0.05, **p ≤ 0.01.

Finally, we examined to what extent Tg*CHRNA5/A3/B4* presented an altered cognitive performance and if so, what was the effect of the chronic nicotine treatment (Figure 5a). In humans, single nucleotide polymorphisms within the *CHRNA5/A3/B4* locus have been associated with reduced performance in cognitive tasks requiring discriminative abilities. In the sense that carriers of the nicotine addiction risk allele present difficulties in working memory and attention and in maintaining and updating information over short delays [27]. In rodents, the hippocampus plays a significant role in discrimination memory [54], while intra-hippocampus infusion of nAChRs agonists facilitate recognition memory [55]. We found that saline-treated Tg*CHRNA5/A3/B4* mice exhibited impaired short-term novelty recognition memory, as compared to WT. During the test session of the novel object recognition paradigm, they showed a similar exploration rate towards the familiar and novel objects as compared to WT (Additional file 3: Figure S3a). As a consequence, the discrimination index was significantly reduced in Tg*CHRNA5/A3/B4* mice, compared to saline-treated WT littermates (*p* = 0.008, Figure 5c). During this recognition session, no differences were detected between genotypes in total exploration rates or anxiety-like behavior (Additional file 3: Figure S3b-c).

Chronic nicotine did not affect novelty recognition memory in WT animals, but it completely reversed the impaired short-term novel object recognition memory in saline-treated Tg*CHRNA5/A3/B4* mice (*p* = 0.035, Figure 5c). The treatment reduced the time that transgenic mice spent exploring the familiar object, while increased the exploration time for the novel object (Additional file 3: Figure S3a). Both WT and Tg*CHRNA5/A3/B4* receiving the drug showed similar amounts of exploration and anxiety-like behavior, as compared to saline-treated mice (Additional file 3: Figure S3b-c).

## Discussion

The present study demonstrates that the *CHRNA5/A3/B4* gene cluster contributes to defining the dendritic complexity of hippocampal pyramidal neurons, the excitatory and inhibitory inputs in the hippocampus and novelty recognition memory. Moreover, deregulation of this genomic region modifies the effects of a chronic nicotine treatment on hippocampal neuroplasticity and cognitive function.

Overexpression of the *CHRNA5/A3/B4* cluster in mice leads to a significant reduction in the dendritic arborization of CA1 pyramidal neurons. Morphological reconstructions using *Lucifer Yellow* injections and genetically driven *Thy-YFP* expression in mice revealed that pyramidal neurons of Tg*CHRNA5/A3/B4* show a reduced total dendritic tree length, volume and branching, resulting in decreased target space. Our morphological analysis also revealed a higher proportion of stubby spines on both apical and basal dendrites in transgenic pyramidal neurons. Stubby spines are considered immature forms of spines in the adult brain [56] and an increased proportion in Tg*CHRNA5/A3/B4* support the view that overexpression of the *CHRNA5/A3/B4* cluster impairs spine maturation. Moreover, immunostaining results for the presynaptic marker VGLUT1 showed that excitatory synaptic inputs in CA1 *strata radiatum* and *oriens* in Tg*CHRNA5/A3/B4* are reduced. Spines lacking synaptic inputs are thought to be less stable in time [57], and thus may explain the increased immature spines in Tg*CHRNA5/A3/B4*. Intriguingly, our results resemble those observed in CA1 pyramidal neurons of mice lacking the α7 nAChR subunit (α7KOs) that show reduced dendritic complexity along with increased dendritic spine density [58]. However, although α7KOs lack glutamatergic synapses in the CA1 region, they show no changes in GABAergic synapses [51]. This contrasts our data, since Tg*CHRNA5/A3/B4* show reduced inhibitory inputs in CA1 *strata radiatum* and *oriens* along with an increased ratio of glutamatergic/GABAergic inputs. Loss of inhibition and hippocampal hyper-excitability has been associated with reduced growth of dendritic arbors [59], and delayed maturation and stability of dendritic spines [16,17]. Noteworthy, the phenotype of Tg*CHRNA5/A3/B4* cannot be attributed to reduced α7-nAChRs since our previous studies showed normal levels of α7-nAChRs on the hippocampus [38]. Interestingly, α3 nAChR subunit is increased in the hippocampus of α7KOs [60], thus suggesting that the phenotype of α7KOs could be interpreted as a consequence of a α3-nAChRs overexpression.

Disrupted dendritic arbor and spines in the CA1 pyramidal neurons usually are assumed to impair synaptic efficacy in this region [61,62]. Nevertheless, electrophysiological recordings demonstrated that applying HFS to the SC afferents elicits similar degree of CA1 synaptic potentiation in transgenic hippocampal slices. The PPR is also similar between genotypes, suggesting no change in release probability. In the CA1 region, nAChRs containing the α5, α3 or β4 subunits are mainly found on *stratum radiatum* interneurons [30,32], where they control pyramidal cell soma and apical dendritic tree excitability. Nevertheless, they are also expressed by pyramidal neurons [33]. nAChRs play a significant role in the expression of LTP in the CA1 [7,63] and it may be possible that increased nAChRs in CA1 region of Tg*CHRNA5/A3/B4* [37] contribute to synaptic efficacy in SC-CA1 connections, overcoming the detrimental structure of CA1 pyramidal neurons.

Because in our transgenic model the overexpression of the cluster *CHRNA5/A3/B4* occurs since fertilization [37], the overall structural and network connectivity alterations detected in Tg*CHRNA5/A3/B4* could arise from early development. Our studies in cultured hippocampal neurons support this idea since transgenic neurons develop with a less complex dendritic tree, higher proportions of immature filopodia-like structures and also deficits in GABAergic inputs. Recent observations have shown an important role of α5-nAChRs during development. Thus, mice lacking the α5 subunit (α5KOs) lose a dendritic pruning of apical dendrites in developing cortical pyramidal neurons [64]. This would suggest that the reduced dendritic branching in Tg*CHRNA5/A3/B4* could be the result of excessive pruning driven by α5-nAChRs. The loss of pruning in α5KOs has been associated with attentional deficits in these mutant mice [64,65]. We found impaired short-term recognition memory in our Tg*CHRNA5/A3/B4* that may also be related with this cognitive role of α5-nAChRs. In humans, genetic variations within the *CHRNA5/A3/B4* cluster are associated with lower discriminative abilities and with an increase in the levels of nAChRs expression [27]. In rodents, the novel object recognition paradigm is the best suited to study discriminative abilities. Performance in the novel object recognition test depends on the hippocampus and the perirhinal cortex, and in fact, the activation of nAChRs in both regions facilitates recognition memory [55]. Moreover, the same genetic variations have also been associated with decreased functional connectivity strength in cortical and subcortical circuits, including the hippocampus, shown by functional magnetic resonance imaging studies [66]. Thus, it may be conceivable that *CHRNA5/A3/B4* overexpression induces alterations in the perirhinal cortex, but this is less probable due to the low levels of expression of the α3, α5 and β4 nAChRs subunits in this region [67,68].

Next we investigated how the *CHRNA5/A3/B4* region influences the effects of nicotine on hippocampal plasticity. Our findings demonstrate that chronic nicotine exposure differentially shapes the dendritic tree of CA1 pyramidal neurons in Tg*CHRNA5/A3/B4* and WT mice. In WT mice, chronic nicotine treatment significantly reduces the number of basal and apical dendrites. Previous studies have observed similar effects on hippocampal morphology but upon developmental exposure to nicotine [69,70]. Remarkably, the effects of nicotine are opposite in Tg*CHRNA5/A3/B4,* in which nicotine increases arborization completely rescuing the reduced dendritic complexity. Similar to our results, in α5KOs, nicotine treatment also compensates their pruning defect and abnormal increase of dendritic branching in developing cortical neurons, while the same treatment causes opposite effects in control animals [71]. This suggests that α5-nAChRs may contribute to the differential response to nicotine observed in Tg*CHRNA5/A3/B4*. Recent studies have demonstrated that genetic factors define different effects of cocaine in modifying the dendritic arbor of CA1 pyramidal neurons. Drug-induced plasticity in dendritic complexity is higher in rats prone to development of drug addiction [72]. Both Tg*CHRNA5/A3/B4* and α5KO mice show increased nicotine self-administration, as compared to control mice [37,73,74]. Whether the differential response to nicotine on dendritic arborization could be linked to development of nicotine addiction requires further investigation.

We also found that chronic exposure to nicotine increases the density of mushroom-like spines in apical dendrites of CA1 pyramidal neurons in both genotypes and compensates for the reduced proportion of stubby versus mushroom-like spines in Tg*CHRNA5/A3/B4*. However, immunostaining experiments showed that chronic nicotine treatment induces differential genotype effects on presynaptic excitatory and inhibitory inputs. In WT mice, nicotine reduces both VGAT and VGLUT1 presynaptic inputs but does not affect the E/I balance. This is in contradiction with previous work reporting that nicotine increases only glutamatergic synapses in the CA1 region, without changing GABAergic synapses [51]. Differences in the dose of nicotine used, duration of the treatment and the age of animals could explain these discrepancies. Conversely, in Tg*CHRNA5/A3/B4* mice, chronic nicotine normalizes the reduced glutamatergic and GABAergic presynaptic inputs and restores the E/I imbalance. The experiments in primary cell cultures confirmed that overexpression of *CHRNA5/A3/B4* alters the effects of nicotine on dendrite branching and presynaptic VGAT puncta. A differential response to GABA in Tg*CHRNA5/A3/B4* mice may require further investigation, given the important role of GABA during the development of nicotine addiction [75].

The electrophysiological recordings revealed that chronic nicotine treatment facilitates LTP induction at SC-CA1 pathway, in agreement with previous published data [76-78]. Nevertheless, this effect was similar in both genotypes. Since nicotine enhances LTP responses via nAChRs containing the β2 subunit [79], it seems likely that this response to nicotine is not altered in our Tg*CHRNA5/A3/B4*.

According to other authors [80], we found that chronic nicotine treatment has no impact on recognition memory in WT animals. Interestingly, the same treatment restores the impairment in recognition memory in Tg*CHRNA5/A3/B4* mice, similar to what has been described in humans with genetic mutations in this cluster [28].

In summary, in this study we provide new evidence that the overexpression of the *CHRNA5/A3/B4* region disrupts pyramidal neuronal structure in the hippocampus, and thus affecting the cognitive capacities. The present work also demonstrates that *CHRNA5/A3/B4* overexpression modifies nicotine-induced changes on dendritic architecture, presynaptic excitatory/inhibitory inputs and recognition memory, but not on synaptic potentiation. Understanding how the *CHRNA5/A3/B4* locus drives the effects of nicotine may help to develop new therapeutic strategies for the pathogenesis of tobacco addiction.

## Additional files

Additional file 1: Figure S1. Morphological analysis of the dendritic tree in *Lucifer Yellow* transfected CA1 pyramidal neurons. The apical dendritic tree of Tg*CHRNA5/A3/B4* (Tg) showed reduced total number of dendrite nodes **(a)**, length **(b)** and volume **(c)**, as compared to wild type (WT) mice (n = 5–10 cells/animal; 4–5 animals/group from ≥ 3 experiments). **p* ≤ 0.05, ****p* ≤ 0.001. Additional file 2: Figure S2. Number of pyramidal somas per area in the CA1 and CA3 layers. *Thy1-yellow fluorescent protein (YFP)-*wild type (WT) and *Thy1-YFP-* Tg*CHRNA5/A3/B4* (Tg) mice that received either saline (Sal) or nicotine (Nic, 3.25 mg/Kg/d) for 7 d showed similar number of pyramidal cell somas per area in *stratum pyramidale* (SP) CA1 and CA3 layers (n = ≥100 images animal; 4–5 animals/group from ≥ 3 experiments). Additional file 3: Figure S3. Novel object recognition test session. Tg*CHRNA5/A3/B4* (Tg) mice spent similar amount of time (s) exploring the familiar and novel objects, as compared to their wild type (WT) littermates. Chronic administration of nicotine (Nic, 3.25 mg/Kg/d) for 5 d increased the time that Tg spent exploring the novel object while reduced the time exploring the familiar object, in comparison to Tg that received saline (Sal) **(a)**. No differences were observed among the four groups of animals in total time of exploration **(b)** and, time spent in the periphery and centre of the open field (%) **(c)** along the 5 min duration session (n = 10–12 animals/group from ≥ 3 experiments).
