# Supplementary figures and images for: Hippocampal changes produced by overexpression of the human CHRNA5/A3/B4 gene cluster may underlie cognitive deficits rescued by nicotine in transgenic mice
Source: Acta Neuropathol Commun. 2014 Nov 11;2:147. doi: 10.1186/s40478-014-0147-1 (PMC4236452; doi:10.1186/s40478-014-0147-1)

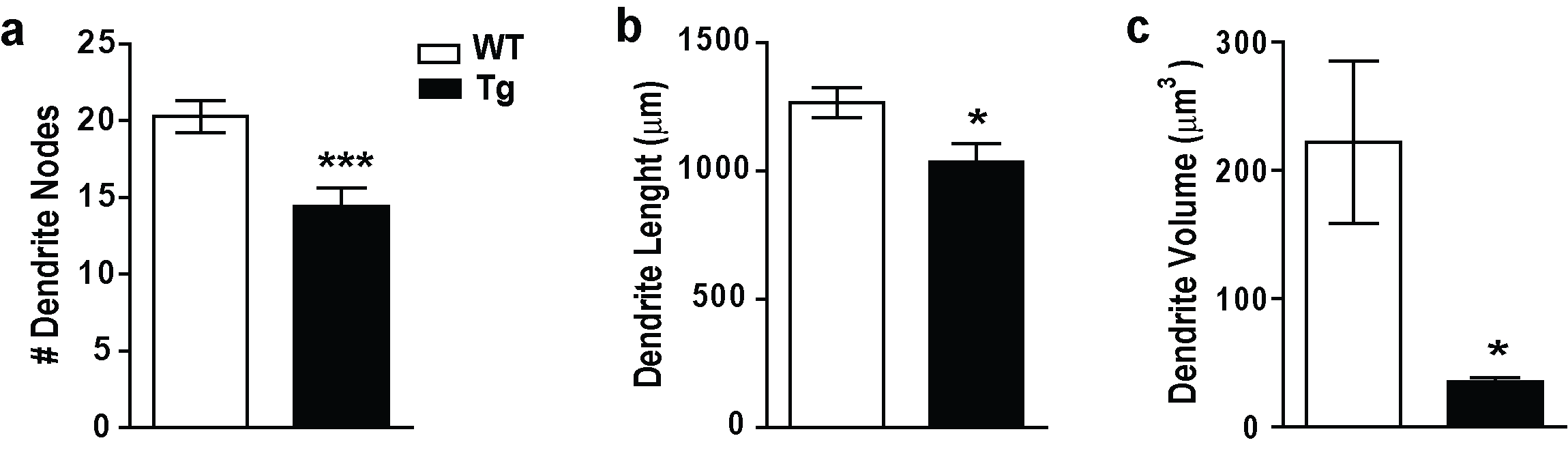

Supplement: Additional file 1: Figure S1. — Morphological analysis of the dendritic tree in Lucifer Yellow transfected CA1 pyramidal neurons. The apical dendritic tree of TgCHRNA5/A3/B4 (Tg) showed reduced total number of dendrite nodes (a), length (b) and volume (c), as compared to wild type (WT) mice (n = 5–10 cells/animal; 4–5 animals/group from ≥ 3 experiments). *p ≤ 0.05, ***p ≤ 0.001. [file 40478_2014_147_MOESM1_ESM.tiff]

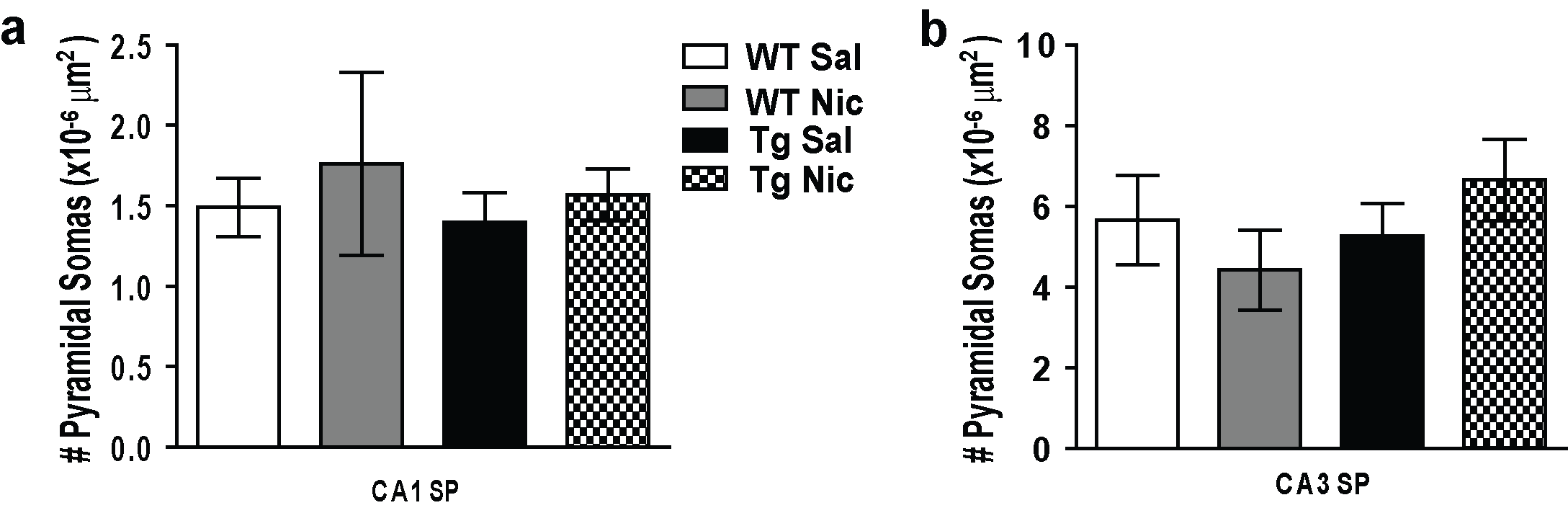

Supplement: Additional file 2: Figure S2. — Number of pyramidal somas per area in the CA1 and CA3 layers. Thy1-yellow fluorescent protein (YFP)-wild type (WT) and Thy1-YFP- TgCHRNA5/A3/B4 (Tg) mice that received either saline (Sal) or nicotine (Nic, 3.25 mg/Kg/d) for 7 d showed similar number of pyramidal cell somas per area in stratum pyramidale (SP) CA1 and CA3 layers (n = ≥100 images animal; 4–5 animals/group from ≥ 3 experiments). [file 40478_2014_147_MOESM2_ESM.tiff]

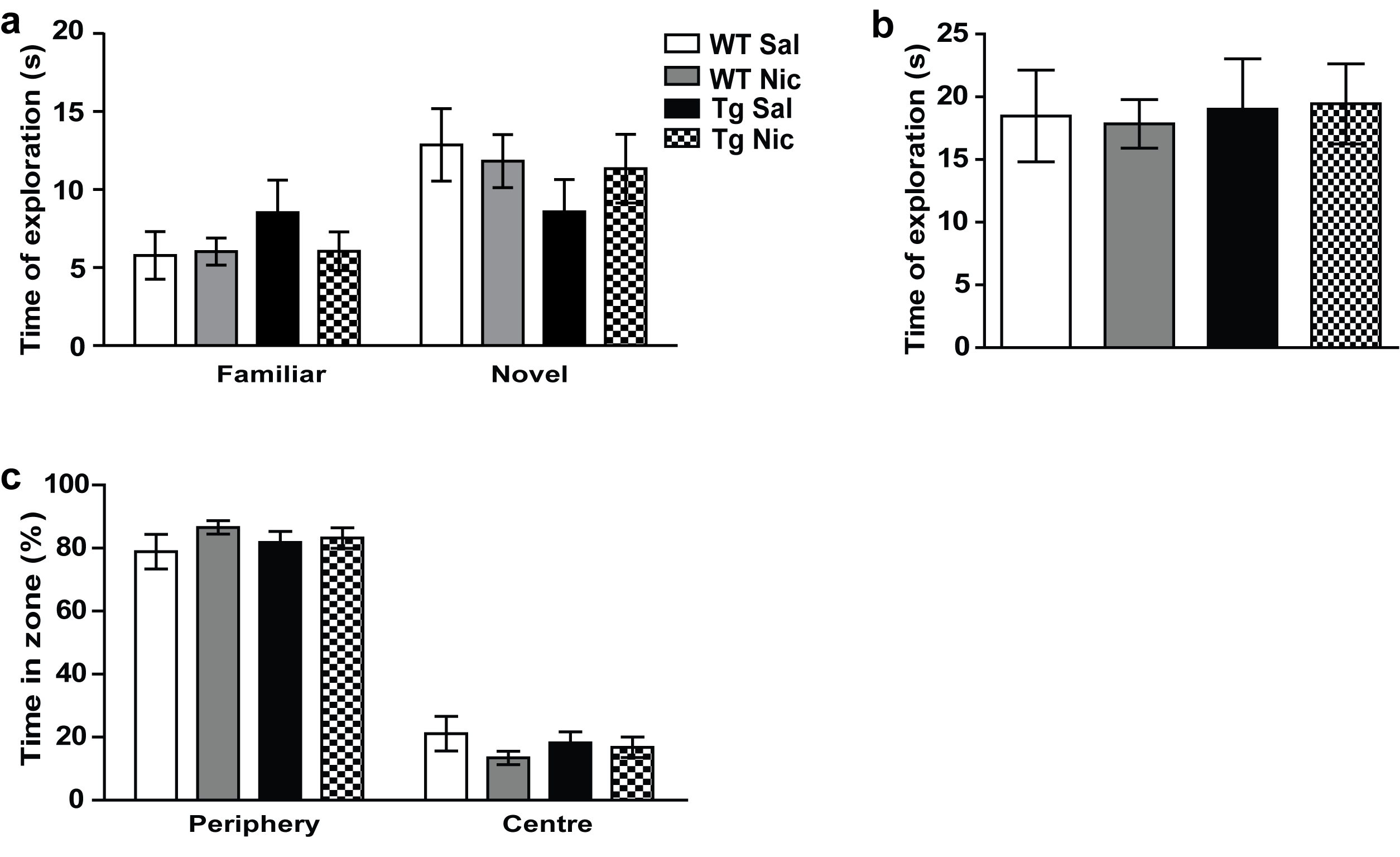

Supplement: Additional file 3: Figure S3. — Novel object recognition test session. TgCHRNA5/A3/B4 (Tg) mice spent similar amount of time (s) exploring the familiar and novel objects, as compared to their wild type (WT) littermates. Chronic administration of nicotine (Nic, 3.25 mg/Kg/d) for 5 d increased the time that Tg spent exploring the novel object while reduced the time exploring the familiar object, in comparison to Tg that received saline (Sal) (a). No differences were observed among the four groups of animals in total time of exploration (b) and, time spent in the periphery and centre of the open field (%) (c) along the 5 min duration session (n = 10–12 animals/group from ≥ 3 experiments). [file 40478_2014_147_MOESM3_ESM.tiff]
